# Etiology, Pathophysiology and Mortality of Shock in Children in Low (Middle) Income Countries: A Systematic Review

**DOI:** 10.1093/tropej/fmac053

**Published:** 2022-07-07

**Authors:** Roxanne Assies, Ilse Snik, Mercy Kumwenda, Yamikani Chimalizeni, Josephine Langton, Job B M van Woensel, Allan Doctor, Job C J Calis

**Affiliations:** Amsterdam UMC location University of Amsterdam, PICU, Emma Children’s Hospital, Meibergdreef 9, Amsterdam, the Netherlands; Amsterdam Centre for Global Child Health, Amsterdam, the Netherlands; Department of Paediatrics and Child Health, Kamuzu University of Health Sciences KUHeS Malawi, Blantyre, Malawi; Amsterdam UMC location University of Amsterdam, PICU, Emma Children’s Hospital, Meibergdreef 9, Amsterdam, the Netherlands; Department of Paediatrics and Child Health, Kamuzu University of Health Sciences KUHeS Malawi, Blantyre, Malawi; Department of Paediatrics and Child Health, Kamuzu University of Health Sciences KUHeS Malawi, Blantyre, Malawi; Department of Paediatrics and Child Health, Kamuzu University of Health Sciences KUHeS Malawi, Blantyre, Malawi; Amsterdam UMC location University of Amsterdam, PICU, Emma Children’s Hospital, Meibergdreef 9, Amsterdam, the Netherlands; Amsterdam Centre for Global Child Health, Amsterdam, the Netherlands; University of Maryland School of Medicine, Baltimore, MD, USA; Amsterdam UMC location University of Amsterdam, PICU, Emma Children’s Hospital, Meibergdreef 9, Amsterdam, the Netherlands; Amsterdam Centre for Global Child Health, Amsterdam, the Netherlands; Department of Paediatrics and Child Health, Kamuzu University of Health Sciences KUHeS Malawi, Blantyre, Malawi

**Keywords:** shock, circulatory insufficiency, low- and middle-income countries, children, pediatric, review

## Abstract

**Objectives:**

Shock is a life-threatening condition in children in low- and middle-income countries (LMIC), with several controversies. This systematic review summarizes the etiology, pathophysiology and mortality of shock in children in LMIC.

**Methods:**

We searched for studies reporting on children with shock in LMIC in PubMed, Embase and through snowballing (up to 1 October 2019). Studies conducted in LMIC that reported on shock in children (1 month–18 years) were included. We excluded studies only containing data on neonates, cardiac surgery patients or iatrogenic causes. We presented prevalence data, pooled mortality estimates and conducted subgroup analyses per definition, region and disease. Etiology and pathophysiology data were systematically collected.

**Results:**

We identified 959 studies and included 59 studies of which six primarily studied shock. Definitions used for shock were classified into five groups. Prevalence of shock ranged from 1.5% in a pediatric hospital population to 44.3% in critically ill children. Pooled mortality estimates ranged between 3.9-33.3% for the five definition groups. Important etiologies included gastroenteritis, sepsis, malaria and severe anemia, which often coincided. The pathophysiology was poorly studied but suggests that in addition to hypovolemia, dissociative and cardiogenic shock are common in LMIC.

**Conclusions:**

Shock is associated with high mortality in hospitalized children in LMIC. Despite the importance few studies investigated shock and as a consequence limited data on etiology and pathophysiology of shock is available. A uniform bedside definition may help boost future studies unravelling shock etiology and pathophysiology in LMIC.

## INTRODUCTION

Shock is a failure of the circulatory system that can complicate many different diseases and is one of the most important mechanisms contributing to pediatric death following respiratory failure [1–4]. Considering the limited preventative and curative healthcare systems and resources in low- and middle-income countries (LMIC) pediatric shock may be more common and associated with an even worse outcome compared to high income countries (HIC).

Fluid boluses are considered the mainstay of shock treatment in HIC. However, the results of the Fluid Expansion as Supportive Therapy (FEAST) trial, challenged this approach in the African setting after demonstrating increased mortality associated with fluid bolus administration. These results remain unexplained and the discussion concerning the generalizability of the FEAST-trial findings and alternative therapeutic approaches feasible for resource-limited settings in LMIC has not meaningfully advanced [5, 6]. In part, this may be explained by our lack of understanding of the etiology and pathophysiology of shock in children in LMIC, which may differ from those in HIC.

Previous systematic reviews have focused on the treatment of shock while the etiology and pathophysiology of shock in children in LMIC has not been systematically reviewed [7–12]. With this review, we specifically wanted to focus on the importance of pediatric shock in LMIC and review the underlying etiology and pathophysiology, as we need a further understanding of this first, before attempting to optimize treatment strategies for shock in children. The aims of this study were to summarize current data regarding the etiology and pathophysiology of pediatric shock, and to assess the prevalence and mortality of shock in children admitted to hospitals in LMIC.

## METHODS

### Search strategy and selection criteria

For this literature review and meta-analysis, we searched PubMed and Embase for studies reporting on shock in children in LMIC published up to 1 October 2019 using the following search terms: (shock OR circulatory failure OR impaired circulation) AND (caus* OR etiolog* OR aetiology* OR pathophysiolog* OR pathophys*) AND (Africa OR Asia OR Caribbean OR South America OR Latin America) AND (Child OR infant OR Paediatric OR Pediatric). Snowballing of references of relevant articles and international guidelines was used to identify additional studies. We excluded literature that was not formally published. We screened articles, firstly by title and abstract, secondly by full text assessment using pre-established inclusion and exclusion criteria. We included studies that reported on shock in children between 1 month and 18 years old and that were conducted in LMIC based on the World Bank classification [13]. We included both interventional and observational studies without language restriction. We excluded case reports, studies that included less than five patients with shock, and reviews without original data, as well as studies focusing on adults, neonates cardiac surgery patients, or shock by an iatrogenic cause only. The screening and data extraction were independently conducted by two reviewers (RA and IS). Any discrepancies were discussed and resolved with a third reviewer (JC).

### Data extraction and quality assessment

Endnote X7 was used to perform screening and remove duplicates. We assessed external validity by reporting study design, sample size, and setting. Measurement and selection bias was assessed by reporting main study population and microbiological diagnostic capacity [14, 15]. We did not exclude studies based on quality to provide a comprehensive overview. Data extraction was performed using a predefined list of variables including country and region, study population, definition of shock used, outcome (mortality), etiology and pathophysiology. We reported our findings in adherence to the PRISMA-guidelines [16].

#### Prevalence and mortality

We presented prevalence as a percentage and 95% confidence interval. We conducted meta-analysis of mortality data using statistical analysis software R v3.6.1 applying the ‘metaprop’ function of the ‘meta’ package. We applied the Freeman-Tukey double arcsine transformation to stabilize data and random effect models (DerSimonain-Laird estimator) to calculate the overall pooled mortality assuming heterogeneity [17, 18]. We report I^2^ and Cochran Q-test to assess heterogeneity. We conducted subgroup analysis per shock definition used, region and etiology using random effect models. We performed sensitivity analyses to assess major differences in overall pooled mortality when excluding studies with no definition for shock or interventional studies and applied different transformation methods (logit, arcsine, log and no transformation) and the generalized linear mixed model [19].

#### Etiology and pathophysiology

Data on etiology and pathophysiology of shock are presented as a narrative synthesis in the discussion. We systematically selected articles concerning etiology and categorized these based on a predefined differential diagnosis for shock (infectious diseases, cardiogenic causes, diarrhea/dehydration, anaphylaxis, neurogenic, trauma, burns and toxic causes). We similarly selected articles describing pathophysiological type of shock.

## RESULTS

**Fig. 1.**
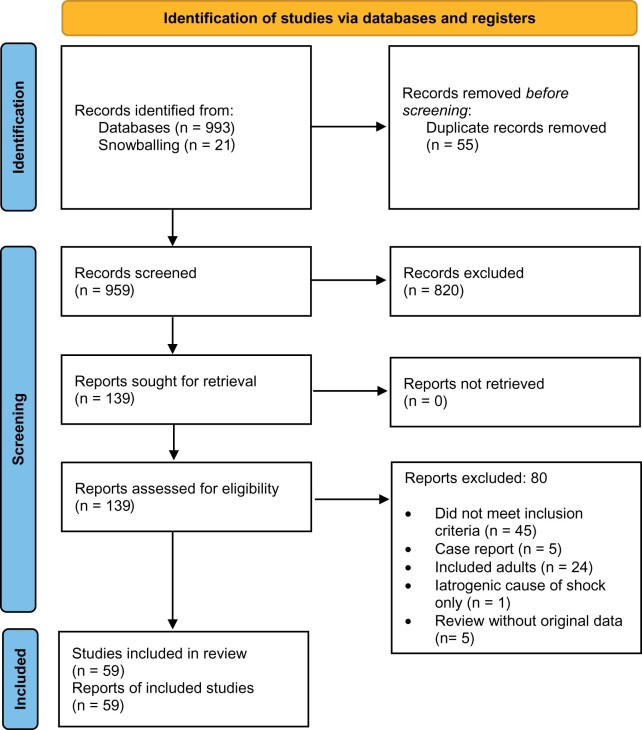
Flow diagram of included studies.

The search resulted in 993 articles and we identified 21 additional articles through snowballing. After removing duplicates 959 articles remained of which 59 studies were included representing 10,250 children with shock (Figure 1). Fifty-three (89.8%) studies reported a definition for shock (Table 1 and 2). We identified different definitions in these studies which we divided in five subgroups (Table 3, Supplementary Table 1). Eighteen (30.5%) studies used a definition from sepsis guidelines, 16 (27.1%) studies used a self-adapted definition of shock and six (10.2%) applied a definition based on the WHO ETAT (Table 3).

**Table 1:** Description of characteristics of included studies that primarily studied shock or included shock with different etiologies (n = 6)

Author, year	Country (region)	Population and setting	Age (yr.)^a^	Test^b^	Study design	Definition of shock	Sample size	Shock (%)	Etiology				
									Gastro- enteritis	Malaria	Sepsis	Severe anemia	Other/comorbidities
Singh *et al.*, 2006 [20]	India (South Asia)	Children with shock admitted to a tertiary care referral hospital	0.1–15	Yes	Prospective, observational	Sepsis guidelines	2274 total admissions	98 (4.3)	46%	n/a	35%	n/a	17% Cardiogenic shock
Ahmad *et al.*, 2010 [21]	Malawi (sub- Saharan Africa)	Children admitted to resuscitation room in a tertiary hospital	1.2^a^	Yes	Prospective, observational	WHO ETAT	583 admissions, resuscitation room	247 (42.4)	n/a	n/a	n/a	n/a	66% HIV+
Akech *et al.*, 2010 [22]	Kenya (sub- Saharan Africa)	Severely malnourished children with shock admitted to a district hospital	0.5–16	Yes	Prospective, interventional	Other	-	61	67%	n/a	33%	Excl.	100% malnutrition^c^
													38% HIV+
													Congenital heart disease excl.
Maitland *et al.*, 2011 [23]	Uganda, Kenya and Tanzania (sub-Saharan Africa)	Children with severe febrile illness admitted to general pediatric wards of six clinical centers	0.2–12	Yes	Prospective, interventional	Other	-	3170					Malnutrition,^c^ trauma, surgery, burns excl.
								Str. A: 3141	Excl.	57%	12%	32%	4% HIV+
								Str. B: 29	Excl.	45%	13%	45%	7% HIV+
Basnet *et al.*, 2014 [24]	Nepal (South Asia)	Children admitted to a PICU	0–16	Yes	Retrospective chart review	Sepsis guidelines	122 PICU admissions	54 (44.3)	n/a	n/a	67%	n/a	33%^d^
Mbevi *et al.*, 2016 [25]	Kenya (sub-Saharan Africa)	Children with shock admitted to 14 hospitals	0.1–5	Yes	Retrospective chart review	Other^e^	42 937 total admissions	622 (1.5)	94%^f^	n/a	6%	n/a	1% HIV+ Malnutrition^c^, surgery and burns excl.

a If no age range was reported in the methods section the median age is reported.

b If any microbiological test was used to confirm a diagnosis.

c Malnutrition refers to children who were severely malnourished.

d Of the children with shock, 33% were associated with other causes including burns, cardiogenic, severe gastro-enteritis and dehydration.

e Mbevi *et al*. also reported the prevalence of shock using two other definitions for shock: WHO shock + signs of dehydration (0.1%) and adapted WHO definition (7.5%).

f Of the children with gastro-enteritis and shock, 99% had a second diagnoses including pneumonia (46%), malaria (33%) and meningitis (13%).

**Table 2:** Description of characteristics of included studies reporting shock as complication of a studied disease in this population (n = 53)

Author, year	Country (region)	Population and setting	Age (years)^a^	Test^b^	Study design	Definition of shock	Sample size	Shock (%)
Dengue hemorrhagic fever (*n* = 13)								
Srivastava *et al.*, 1990 [26]	India (South Asia)	Children with dengue hemorrhagic fever admitted to a hospital	3–11	Yes	Prospective, observational	WHO DHF	24	17 (70.8)
Bethell *et al.*, 1998 [27]	Vietnam (East Asia and Pacific)	Children with dengue hemorrhagic fever admitted to a pediatric hospital	Median age 6	Yes	Prospective, observational	WHO DHF	443	259 (58.5)
Dung *et al.*, 1999 [28]	Vietnam (East Asia & Pacific)	Children with dengue shock syndrome admitted to a PICU	1–15	Yes	Prospective, interventional	WHO DHF	–	50
Ngo *et al.*, 2001 [29]	Vietnam (East Asia & Pacific)	Children with dengue shock syndrome admitted to a PICU	1–15	Yes	Prospective, interventional	WHO DHF	–	222
Wills *et al.*, 2002 [30]	Vietnam (East-Asia & Pacific)	Children with dengue shock syndrome admitted to a PICU	1–15	Yes	Prospective, observational, also enrolled in interventional study	WHO DHF	–	167
Kabilan *et al.*, 2005 [31]	India (South Asia)	Children with dengue hemorrhagic fever admitted to a pediatric hospital	<15	Yes	Prospective, observational	WHO DHF	143	34 (23.8)
Ranjit *et al.*, 2005 [32]	India (South Asia)	Children with dengue shock syndrome admitted to a PICU	Median age 1.7 and 2.0	Yes	Retrospective chart review	WHO DHF	–	172
Wills *et al.*, 2005 [33]	Vietnam (East Asia & Pacific)	Children with dengue shock syndrome admitted to a PICU	2–15	Yes	Prospective, interventional	WHO DHF	–	512
Pham *et al.*, 2007 [34]	Vietnam (East Asia & Pacific)	Children with dengue hemorrhagic fever admitted to a pediatric hospital	1–15	Yes	Prospective, observational	WHO DHF	–	40
Kamath *et al.*, 2006 [35]	India (South Asia)	Children with dengue hemorrhagic fever admitted to a PICU	<15	Yes	Retrospective chart review	WHO DHF	858	73 (8.5)
Djamiatun *et al.*, 2012 [36]	Indonesia (East Asia & Pacific)	Children with dengue hemorrhagic fever admitted to the pediatric ward or intensive care unit of a university hospital	3–14	Yes	Prospective observational	WHO DHF	73	30 (41.1)
Ngwe Tun *et al.*, 2013 [26]	Myanmar (East Asia & Pacific)	Children with dengue hemorrhagic fever admitted to two hospitals.	≤ 12	Yes	Prospective, observational	WHO DHF	160	12 (7.5)
Pothapregada *et al.*, 2015 [38]	India (South Asia)	Children with dengue hemorrhagic fever admitted to a tertiary care hospital	0–12	Yes	Retrospective chart review	WHO DHF	261	102 (39.1)
Sepsis/septic shock (*n* = 14)								
Upadhyay *et al.*, 2005 [39]	India (South Asia)	Children with septic shock admitted to tertiary care hospital/PICU	0.1–12	Yes	Prospective, interventional	Sepsis guidelines	–	60
Baranwal *et al.*, 2007 [40]	India (South Asia)	Children with disseminated staphylococcal disease admitted to a PICU	0.1–12	Yes	Retrospective chart review	No definition	53	28 (52.8)
Santhanam *et al.*, 2008 [41]	India (South Asia)	Children with septic shock admitted to a PICU	0.1–12	Yes	Prospective, interventional	Sepsis guidelines	–	147
Menif *et al.*, 2009 [42]	Tunisia (Middle East & North Africa)	Children with septic shock admitted to a PICU	0.1–11	Yes	Retrospective chart review	Sepsis guidelines	2487 total admissions	70 (2.8)
Valoor *et al.*, 2009 [43]	India (South Asia)	Children with septic shock admitted to a PICU	0.2–12	Yes	Prospective, interventional	Sepsis guidelines	–	38
Chopra *et al.*, 2011 [44]	India (South Asia)	Children with septic shock admitted to a PICU	2–12	Yes	Prospective, interventional	Sepsis guidelines	–	60
Khan *et al.*, 2012 [45]	Pakistan (South Asia)	Children with sepsis, severe sepsis and septic shock admitted to a PICU	0.1–14	Yes	Retrospective chart review	Sepsis guidelines	133 sepsis admissions	95 (71.4 of sepsis admissions, 12.4 of total admissions)
							767 total admissions	
Couto *et al.*, 2013 [46]	Liberia (sub-Saharan Africa)	Patients admitted to pediatric secondary-care hospital and died	0–15	Yes	Retrospective chart review	Sepsis guidelines	331 with infectious disease, 8254 total admissions	106 (32.0 of infectious disease, 1.3 of total admissions)
Ranjit *et al.*, 2013 [47]	India (South Asia)	Children admitted to a PICU with fluid refractory septic shock	0.3–15	Yes	Retrospective, observational	Sepsis guidelines	–	22
Ranjit *et al.*, 2014 [48]	India (South Asia)	Children admitted to two PICU’s with fluid refractory septic shock	0.1–16	Yes	Prospective, observational	Sepsis guidelines	–	48
Ibrahiem *et al.*, 2016 [49]	Egypt (Middle East & North Africa)	Children with severe sepsis or septic shock admitted to two PICU’s	Median age 1.5	Yes	Prospective, observational	Sepsis guidelines	57	18 (31.6)
Ramaswamy *et al.*, 2016 [50]	India (South Asia)	Children with fluid refractory hypotensive septic shock admitted to a PICU	0.3–12	Yes	Prospective, interventional	Sepsis guidelines	–	60
Kortz *et al.*, 2017 [51]	Bangladesh (South Asia)	Children with (severe) sepsis admitted to a large non-governmental hospital	0.1–5	Yes	Retrospective, cohort study	Sepsis guidelines	328	84 (25.6)
El-Nawawy *et al.*, 2018 [52]	Egypt (Middle East & North Africa)	Children with septic shock admitted to a PICU	0.1–11	Yes	Prospective, interventional	Sepsis guidelines	–	90
Malaria (*n* = 9)								
Maitland *et al.*, 2003 [53]	Kenya (sub-Saharan Africa)	Children with severe malaria admitted to a district hospital	<16	Yes	Retrospective chart review	Other	372	212 (56.9)
Maitland *et al.*, 2005 [54]	Kenya (sub-Saharan Africa)	Children with severe malarial anemia admitted to a district hospital	≥ 0.2	Yes	Prospective, interventional	Other	61	41 (67.2)
Akech *et al.*, 2006 [55]	Kenya (sub-Saharan Africa)	Children with severe malaria, metabolic acidosis and clinical features of shock admitted to a district hospital	≥ 0.3	Yes	Prospective, interventional	Other	–	88
Dondorp *et al.*, 2010 [56]	Mozambique, The Gambia, Ghana, Kenya, Tanzania, Nigeria, Uganda, Rwanda and Democratic Republic of the Congo (sub-Saharan Africa)	Children with severe malaria admitted to 11 centers in 9 countries	<15 years	Yes	Prospective, interventional	Other	5425	662 (12.2)
Akech *et al.*, 2010 [57]	Kenya (sub-Saharan Africa)	Children with severe malaria and metabolic acidosis admitted to a district hospital	≥ 0.5	Yes	Prospective, interventional	Other	–	79
Yadav *et al.*, 2012 [58]	India (South Asia)	Children with severe malaria admitted to a tertiary care hospital	<18	Yes	Retrospective chart review	Other	210	7 (3.33)
Kalinga *et al.*, 2012 [59]	Tanzania (sub-Saharan Africa)	Children with severe malaria admitted to two district hospitals	0.1–12	Yes	Prospective, observational	Other	409 malaria patients	158 (38.6 of malaria patients, 2.7 of total admissions)
							5753 total admissions	
Gehlawat *et al.*, 2013 [60]	India (South Asia)	Children with severe malaria admitted to a tertiary care hospital	0.1–14	Yes	Prospective, observational	Other	35	5 (14.3)
Boyce *et al.*, 2018 [61]	Uganda (sub-Saharan Africa)	Children with malaria presenting at a primary health center	<15	Yes	Prospective, observational	Other	85 severe malaria patients	18 (21.2 of severe malaria, 2.0 of malaria patients)
							914 malaria patients	
Diarrhea (*n* = 6)								
Sarmin *et al.*, 2014 [62]	Bangladesh (South Asia)	Children with diarrhea and severe sepsis admitted to the ICU of Dhaka Hospital of the International Centre for Diarrheal Diseases Research	0–5	Yes	Retrospective chart review	Sepsis guidelines	204	88 (43.1)
Breurec *et al.*, 2016 [63]	Central African Republic (sub-Saharan Africa)	Children with diarrhea admitted to a pediatric hospital	0–5	Yes	Prospective, observational	No definition	333 (cases)	216 (64.8)
Chisti *et al.*, 2017 [64]	Bangladesh (South Asia)	Children with diarrhea admitted to a PICU	0–5	Yes	Retrospective chart review	Sepsis guidelines	219	48 (21.9)
Obonyo *et al.*, 2017 [65]	Kenya, Uganda (sub-Saharan Africa)	Severely malnourished children with diarrhea and (hypovolemic) shock admitted to a district or referral hospital	0.5–5	Yes	Prospective, observational	WHO ETAT	–	20
Akech *et al.*, 2018 [66]	Kenya (sub-Saharan Africa)	Children with diarrhea admitted to 13 first referral-level hospitals	0.1–4.9	No (malaria testing only)	Retrospective, observational	Other	8563	431 (5.0) clinical shock
								37 (0.4)
								WHO shock+ dehydration
Talbert *et al.*, 2019 [67]	Kenya (sub-Saharan Africa)	Children with diarrhea admitted to a district hospital	0.2–4.9	Yes	Retrospective, observational	WHO ETAT	2626 diarrhea patients	55 (2.1 of diarrhea patients, 0.3 of total admissions)
							17 442 total admissions	
Scrub typhus (*n* = 3)								
Kumar *et al.*, 2012 [68]	India (South Asia)	Children with scrub typhus admitted to a tertiary care hospital	1.5–12	Yes	Prospective, observational	No definition	35	12 (34.3)
Palanivel *et al.*, 2012 [69]	India (South Asia)	Children with scrub typhus admitted to a children’s referral hospital	<12	Yes	Prospective, observational	No definition	67	30 (44.8)
Narayanasamy *et al.*, 2016 [70]	India (South Asia)	Children with scrub typhus admitted to a tertiary care hospital	0.5–12	Yes	Prospective, observational	No definition	117	23 (19.7)
Trauma and/or Burns (*n* = 3)								
Nguyen *et al.*, 2002 [71]	Vietnam (East Asia & Pacific)	Children with burns admitted to the National Burn Institute	<15	No	Retrospective chart review	Other	695	–
Osifo *et al.*, 2012 [72]	Nigeria (sub-Saharan Africa)	Children with trauma and/or burns admitted to a teaching hospital (level 1 trauma center) and died	<18	No	Retrospective chart review	No definition	78	33 (42.3)
Patregnani *et al.*, 2012 [73]	Iraq and Afghanistan (Middle East & North Africa)	Children with trauma and/or burns admitted to combat support hospitals	<18	No	Retrospective chart review	Other	744	285 (38.3)
Severe anemia (*n* = 3)								
Pedro *et al.*, 2010 [74]	Kenya (sub-Saharan Africa)	Children with severe anemia admitted to a district hospital	<13	Yes	Retrospective chart review	Other	2265 severe anemia patients, 36 621 total admissions	442^c^ (19.5 of severe anemia patients, 1.2 of total admissions)
Maitland *et al.*, 2019 [75]	Uganda and Malawi (sub-Saharan Africa)	Children with (uncomplicated) severe anemia admitted to four hospitals	0.2–12	Yes	Prospective, interventional	WHO ETAT	787^d^	112 (14.2)
Maitland *et al.*, 2019 [76]	Uganda and Malawi (sub-Saharan Africa)	Children with severe anemia admitted to four hospitals	0.2–12	Yes	Prospective, interventional	WHO ETAT	3196	1058 (33.1)
Chikungunya (*n* = 1)								
Sharma *et al.*, 2018 [77]	India (South Asia)	Children with chikungunya admitted to HDU/PICU	<16	Yes	Retrospective chart review	Sepsis guidelines	49	11 (22.4)
Pneumonia (*n* = 1)								
Webb *et al.*, 2012 [78]	Kenya (sub-Saharan Africa)	Children with pneumonia admitted to a district hospital	0.2–4.9	Yes	Prospective, observational	WHO ETAT	568	43 (7.6)

a If no age range is reported in methods, the median age is reported.

b If any microbiological test was used to confirm a diagnosis.

c Number excluding neonates.

d Control patients only as the children with (uncomplicated) severe anemia who received a blood transfusion are also included in the accompanying paper by Maitland *et al.*, 2019.

**Table 3:** Type of definition for shock used in included studies. The full descriptions of definitions used in each study and how we grouped them are provided in Supplementary Table S1

Type of definition	Number of studies (*N* = 59)	Number of studies reporting mortality (*n* = 43)
	*n* (%)	*n* (%)
Sepsis guidelines	18 (30.5)	14 (32.6)
WHO Dengue (up to 2011)	13 (22.0)	9 (20.9)
WHO ETAT (2016)	6 (10.2)	4 (9.3)
Other definition	16 (27.1)	12 (27.9)
No definition	6 (10.2)	4 (9.3)

Twenty-five studies (42.4%) were conducted in South Asia, 21 (35.6%) in sub-Saharan Africa, nine (15.3%) in East Asia & Pacific and four (6.8%) in the Middle East & North Africa, representing 16 countries in total (Table 1 and 2) [20–78].

In six (10.2%) of the included studies, patient enrolment focused on children with shock or included children with shock with different etiologies (referred to as *studies that primarily studied shock*), two of these studies were conducted in a critical care setting (Table 1). The other 53 (89.8%) studies focused on a specific disease other than shock and reported shock as a complication of this disease. Twenty-one of these studies were conducted in a critical care setting (e.g. shock complicating malaria or diarrhea, Table 2).

### Prevalence

Of the six studies that primarily studied shock, two reported prevalence of shock in a general hospital population of 1.5% (95%-CI: 1.3-1.6%) and 4.3% (95%-CI: 3.4-5.1%) in Kenya and India respectively (Table 1). Two other studies reported prevalence of shock in a critical care setting of 42.4% (95%-CI: 38.4-46.4%) (Malawi) and 44.3% (95%-CI: 35.5-53.1%) (Nepal). No data on the incidence or prevalence of shock in the general population were identified.

### Mortality

**Fig. 2.**
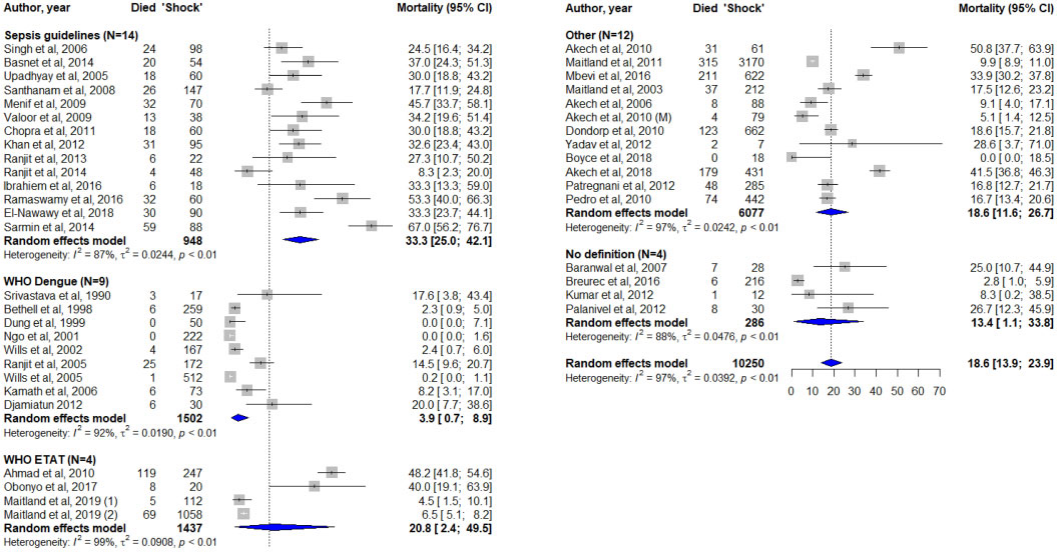
Forest plot including all studies reporting mortality, subgroup analyses for different definitions used in these studies and the overall pooled mortality estimate.

Forty-three (72.9%) studies reported mortality data on children with shock, of which the pooled mortality estimate was 18.6% (95%-CI: 13.9-23.9%), this was a random effects estimate as the dataset had considerable heterogeneity (I^2^ = 97%, Figure 2). In subgroup analyses of mortality for different definitions for shock, we found that studies applying sepsis guidelines definitions demonstrated the highest pooled mortality of 33.3% (95%-CI: 25.0-42.1%). Studies applying the WHO Dengue definition for shock demonstrated the lowest pooled mortality 3.9% (95%-CI: 0.7-8.9). The studies that used (a variation of) the WHO ETAT definition had a pooled mortality of 20.8% (95%-CI: 2.4-49.5%). In the six studies that primarily studied shock, the pooled mortality was 32.8% (95%-CI: 16.4-51.6%). Subgroup analysis per etiology showed high pooled mortality in sepsis 30.3% (95%-CI: 23.1-38.0%) and diarrhea 34.3% (95%-CI: 6.8–69.1%, Supplementary Figure 1). In subgroup analyses per region we found the highest pooled mortality of 38.1% (95%-CI: 29.5-47.0%) in Middle East & North Africa and lowest in East Asia & Pacific 1.3% (95%-CI: 0.0-4.0%, Supplementary Figure 2).

## DISCUSSION AND NARRATIVE REVIEW

Few studies reported the prevalence of shock in children in LMIC, ranging from 1.5% in a general pediatric hospital population up to 44% in children in a critical care setting. The pooled mortality per shock definition ranged from 4-33% and the pooled mortality estimate of the studies that primarily examined shock (*n* = 6) was 33%. We will first discuss these findings and next summarize the data on etiology and pathophysiology of shock in these children.

The prevalence of shock is given its severity common in the general setting and very common amongst critically ill children in LMIC. The prevalence varies from 1.5-4.3% in a general hospital unit to >40% in critically ill children. Although prevalence data were limited to only four studies from LMIC, these data suggest shock is a common clinical presentation in children in LMIC.

We further identified that a fifth of children with shock died during hospital admission. The actual mortality may even be higher, since in the six studies that primarily studied shock the pooled mortality estimate was 33%. This number may reflect the overall chances of survival more accurately, as these studies used shock as a screening diagnosis and results are therefore not biased by underlying diagnoses which may have different outcomes. The heterogeneity of this overall pooled estimate is high and could not simply be explained by shock definition used, etiology or region. This may underline the complexity of shock, which is a well-recognized clinical presentation, but can be caused by very different diseases and underlying pathophysiological mechanisms. Furthermore, contextual factors such as facility diagnostic and therapeutic resources, delayed patient presentation to hospital, and patient access to care vary between the different settings and study designs, and contribute to the heterogeneity of shock mortality in the included studies. Despite the heterogeneity of the pooled mortality estimates, we conclude that children with shock in LMIC have a poor outcome.

Interpretation of the prevalence and mortality data presented in this paper is limited by the inconsistency in shock definitions applied. We found that five subgroups of shock definitions were used, representing 18 different definitions. This is a major source of selection bias, introduces heterogeneity in the pooled mortality estimates and limits external validity of results beyond each specific study setting. Only few studies applied the WHO definition of shock [21, 25, 65]. Although this definition of shock is based on bedside clinical parameters, its validity is criticized as it reflects a very advanced or even irreversible stage of shock which has a mortality of up to 100% [21, 25, 79, 80]. In order to improve our understanding of pediatric shock, a similarly simple, but clinically useful definition or simple bedside diagnostic technique to timely detect (imminent) shock is urgently needed.

### Etiology

Understanding the etiology of pediatric shock in LMIC is essential to develop evidence-based treatment protocols which are especially important in settings with limited diagnostic capacity. The six studies that primarily studied shock did provide some data, however in most studies, diagnostic resources (including microbiological testing) were limited. Comparing these etiology data to those in HIC, some differences can be identified. Firstly, the differential diagnosis of shock in LMIC is broader and includes diseases such as malaria, severe anemia and dengue hemorrhagic fever. Other etiologies that may be more prominent in LMIC include trauma [81] and burns [82]. Secondly, children with shock in LMIC often seem to have several concurrent diagnoses and thus adequate treatment of shock in children in LMIC may require multiple therapies. Thirdly, a very large proportion (46-94%) of children with shock in LMIC had gastrointestinal fluid losses. Lastly, comorbidities such as HIV and malnutrition are more prevalent, all of which were associated with a high mortality and may need to be actively screened for and treated appropriately.

### Differential diagnosis

*Sepsis* is a commonly reported cause of shock in LMIC and had a high pooled mortality (30%). Pneumonia was reported to be the primary underlying diagnosis in studies from LMIC [39, 41, 43, 47–49]. The pathogens reported in septic shock studies predominantly identified gram-negative bacteria (Supplementary Figure 3). These data, however, were mostly from PICU’s in South Asia. Data from other regions and settings are lacking, in part due to limited diagnostic resources, and although gram-negative bacteria are most commonly reported in LMIC, these data may show different pathogens as gram-positive pathogens are more commonly reported in pediatric wards in Africa compared to Asia. More recent concerns further include antimicrobial resistance rates that appear to be increasingly more common and may contribute to an increased prevalence and worse outcome of pediatric shock in the future [83].

*Malaria* may be complicated by shock and in *Plasmodium falciparum,* this may occur in up to 57% of hospitalized children with severe malaria (Table 1). The underlying pathophysiology of malaria associated shock remains unclear. Possible explanations include the inflammatory response to *P. falciparum* and the attributive effect of malaria related complications such as severe anemia and (non-Typhoid Salmonella) bacteremia, all of which are also predictors of poor outcome [53–55, 57, 60, 84].

*Gastroenteritis and diarrhea* was reported in 46-94% of children with shock [20, 25]. Whether these children have gastroenteritis or gastrointestinal symptoms due to systemic illness is less clear. Studies did not report results on gastrointestinal pathogens and sepsis or malaria are often reported as coinciding diagnoses [62, 65, 66]. The pooled mortality in this group was high (30%), however the range of reported mortality was 3 to 67% which may be explained by the (missed) underlying disease such as sepsis. The WHO guidelines on how to treat children with severe dehydration due to gastro-enteritis has become complex, as it requires quick assessment of fluid status, nutritional status and ideally hemoglobin to decide the amount and type of fluid, which in practice can often be challenging [1, 23]. The effect of this algorithmic approach has not been comprehensively evaluated [85, 86].

*Severe anemia* (hemoglobin < 5 g/dL) significantly reduces oxygen delivery capacity, contributes to shock development and was reported in a third of shocked African children [23]. Severe anemia was associated with the highest excess mortality after fluid bolus and WHO guidelines now prioritize blood transfusion as fluid therapy in children with severe anemia and shock, if available, and to give maintenance fluids only until blood is available [1]. Severe anemia is commonly associated with diseases such as malaria, HIV and bacteraemia [74–76, 87]. These findings together suggest that in LMIC, especially in sub-Saharan Africa, severe anemia is a condition that needs to be tested for, treated and considered also in the context of other causes of shock.

*Dengue hemorrhagic fever* is common in Asia and was reported to have a relatively better prognosis than other causes of shock [88]. Dengue virus can directly cause shock but may also be complicated by gastrointestinal bleeding [27, 35, 36, 38] which is associated with increased mortality [30]. Bacterial co-infections appear to be uncommon [26, 35].

### Pathophysiology

Timely and adequate identification of the underlying pathophysiological mechanism of shock is essential to appropriately select the different potential lifesaving therapies [1, 2]. Shock pathophysiology may be very different in children in LMIC as compared to HIC, which is indirectly suggested by the detrimental effects of fluid boluses in African children [23].

In our dataset only one study described the contribution of different pathophysiological types of shock in children, but had limitations as these findings were based on physical exam only [20]. Seven additional studies reported data on the pathophysiology of shock in subgroups of children using ultrasound techniques [32, 35, 47, 48, 52, 66, 89]. The data from these studies suggest that hypovolemia is the most common mechanism of shock in children in LMIC, which may not be surprising, considering the high prevalence of gastroenteritis complaints in these children [1]. Hypovolemia may further be caused by increased capillary leakage as occurs in inflammatory processes and in children with dengue shock syndrome [33]. The role of hypovolemia in sepsis and malaria is, however, less clear. Pathophysiological data based on ultrasound findings supported that hypovolemia was common in children with malaria [89]. However, these findings are in contrast to the FEAST-trial results which showed that boluses of IV fluids were associated with excess mortality, also in children with malaria and sepsis with shock [23]. Despite several efforts by the FEAST-trial authors and subsequent studies, the complex interplay between sepsis, malaria and hypovolemia remains unclear. The FEAST-trial authors report circulatory collapse as main terminal clinical events and increase in mortality after, not before or during, fluid bolus. They report that these findings may support the hypothesis of “re-perfusion” injury after fluid bolus, leading to organ damage and “myocardial stunning” [5, 90]. Levin *et al*. however conclude that adverse effects of fluid boluses were more likely associated with respiratory and neurological dysfunction, hyperchloremic acidosis and reduction in hemoglobin concentration [91]. Long *et al*. reported that median blood pressure initially decreased after fluid bolus, and returned to baseline after one hour in Australian children with sepsis [92]. To date the FEAST trial is the most convincing interventional study, but the unexpected results, the underlying pathophysiological mechanism and (adverse) effects of fluid bolus remain poorly understood.

The role of cardiogenic shock may be important in LMIC. Congenital heart disease was found as an important underlying cause in India [20]. Cardiac dysfunction was described in septic shock [47, 48, 52], malaria [89] and dengue [32, 35]. Although cardiac dysfunction has long been assumed to be present in severely malnourished children, this could not be confirmed in echocardiography studies [66, 93]. Continuous adrenaline infusion has been successfully used as inotropic therapy in children in LMIC with cardiogenic dysfunction, but reliable detection and monitoring may be an issue in these settings [47, 48, 50, 52].

Whilst distributive and obstructive shock appear to be uncommon in LMIC [20], although this could also be due to limited diagnostic resources, dissociative shock (severe anemia) may very important. Severe anemia affects a third of shocked children in sub-Saharan Africa, complicates fluid therapy and is associated with a decreased survival [23].

This review has several other limitations, apart from the previously discussed heterogeneity of included studies, different definitions of shock applied and limited number of studies that primarily studied shock. Overrepresentation of some countries, critical care settings (PICU’s) and diseases may have introduced selection and information bias. Furthermore, limited diagnostic resources in LMIC have contributed to the lack of data on etiology and pathophysiology of shock. A syndromic approach may therefore be more appropriate in settings where diagnostic resources are limited, and underlying diagnoses are not (quickly) apparent. Together, these limitations underline that despite the high prevalence and mortality, there is a lack of data on shock in children in LMIC.

In conclusion, we found that shock is a common clinical problem in hospitalized children in LMIC affecting nearly half of those critically ill and associated with a very high mortality. Despite the importance, only very few studies focused on shock in this population. The etiologies of shock in LMIC include gastroenteritis, sepsis, malaria and severe anemia and often coincide. The limited data on the pathophysiology suggest that besides hypovolemia, cardiac dysfunction and dissociative shock, or a combination of pathophysiological mechanisms, are important in LMIC. In order to improve the outcome of shock in children in LMIC, we first need to develop a reliable and valid bedside definition for shock and gain comprehensive data on shock etiology and pathophysiology.

## Supplementary Material

fmac053_Supplementary_Data
