# Supplementary material for: Etiology, Pathophysiology and Mortality of Shock in Children in Low (Middle) Income Countries: A Systematic Review
Source: J Trop Pediatr. 2022 Jul 7;68(4):fmac053. doi: 10.1093/tropej/fmac053 (PMC9586536; doi:10.1093/tropej/fmac053)
Supplement: fmac053_Supplementary_Data [file fmac053_supplementary_data.zip › 20220505 Supplementary Figure 3 Blood Culture Results.pptx]

## Slide 1
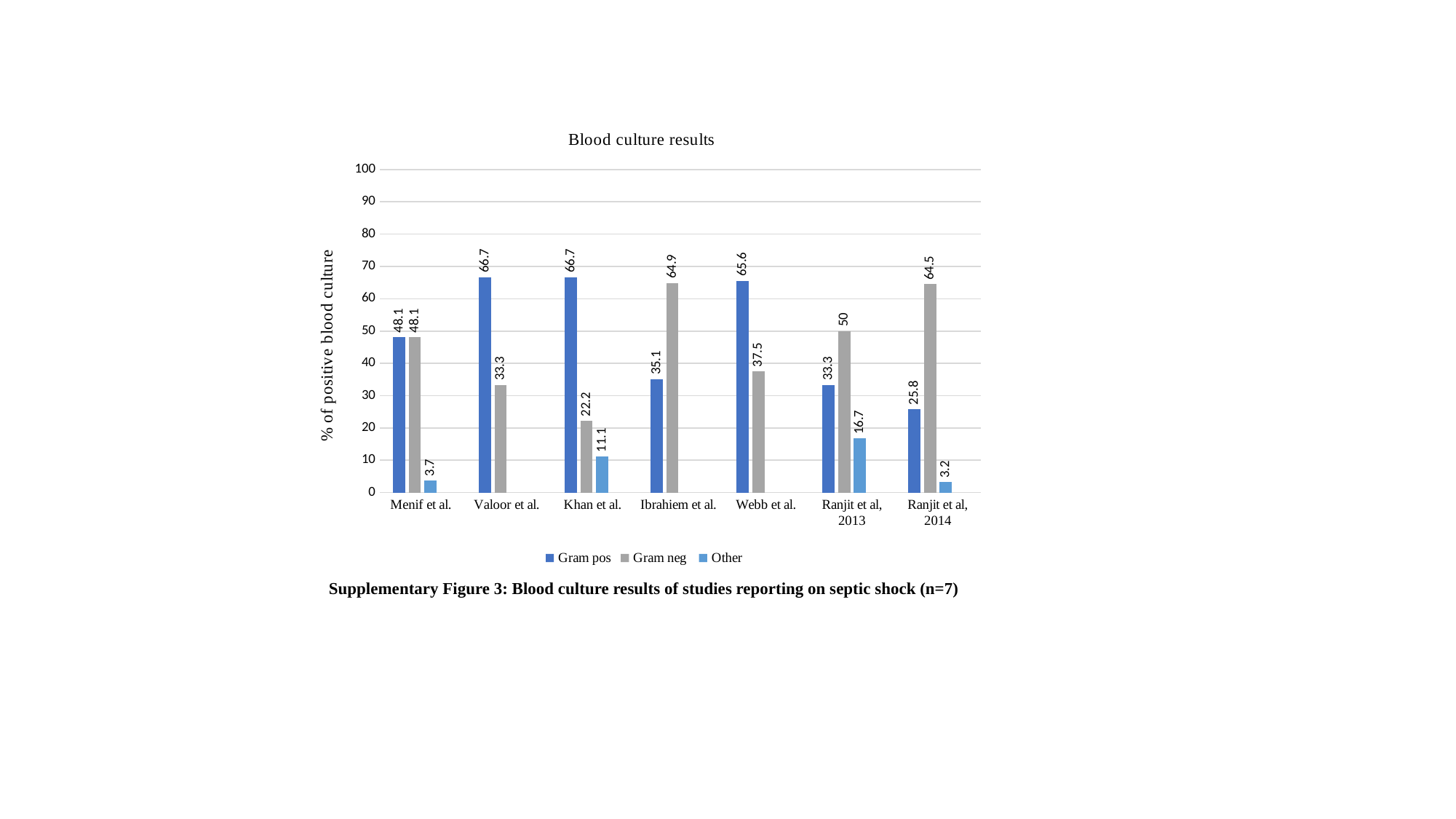

### Chart: Blood culture results
| Category | Gram pos | Gram neg | Other | |
|---|---|---|---|---|
| Menif et al. | 48.1 | 48.1 | 3.7 | None |
| Valoor et al. | 66.7 | 33.3 | None | None |
| Khan et al. | 66.7 | 22.2 | 11.1 | None |
| Ibrahiem et al. | 35.1 | 64.9 | None | None |
| Webb et al. | 65.6 | 37.5 | None | None |
| Ranjit et al, 2013 | 33.3 | 50.0 | 16.7 | None |
| Ranjit et al, 2014 | 25.8 | 64.5 | 3.2 | None |Supplementary Figure 3: Blood culture results of studies reporting on septic shock (n=7)
