# Supplementary material for: Etiology, Pathophysiology and Mortality of Shock in Children in Low (Middle) Income Countries: A Systematic Review
Source: J Trop Pediatr. 2022 Jul 7;68(4):fmac053. doi: 10.1093/tropej/fmac053 (PMC9586536; doi:10.1093/tropej/fmac053)
Supplement: fmac053_Supplementary_Data [file fmac053_supplementary_data.zip › 20211215 Supplementary Table 1 Definitions of shock.docx]

**Supplementary Table 1: Description of definitions for shock used in included studies (N=59)**

|  | Details of definition (in reference or in paper) | Number of studies (N=59) |
| --- | --- | --- |
| 1. Sepsis Guidelines | | |
| ACCCM | “The clinical diagnosis of septic shock is made in children who have a suspected infection manifested by hypothermia or hyperthermia and have clinical signs of decreased perfusion, including decreased mental status, prolonged capillary refill of > 2 secs (cold shock) or flash capillary refill (warm shock), diminished (cold shock) or bounding (warm shock) peripheral pulses, mottled cool extremities (cold shock), or decreased urine output of <1 mL/kg/hr. Hypotension is not necessary for the clinical diagnosis of septic shock; however, its presence in a child with clinical suspicion of infection is confirmatory.” | 6  Menif et al.  Couto et al.  Singh et al.  Ranjit et al, 2013  Ranjit et al, 2014  El-Nawawy et al. |
| Surviving Sepsis Campaign (excluding SSC 2020)  Goldstein et al. | “Children and neonates maintain higher vascular tone than adults. Therefore, the shock state occurs long before hypotension in children. Septic shock in pediatric patients is defined as a tachycardia (may be absent in hypothermic patient) with signs of decreased perfusion including decreased peripheral pulses compared to central pulses, altered alertness, flash capillary refill or capillary refill >2 secs, mottled or cool extremities, or decreased urine output. Hypotension (in children less than 2 SD below normal for age) is a sign of late and decompensated shock in children” (Surviving Sepsis Campaign)  “Septic shock: sepsis and cardiovascular organ dysfunction, defined as: despite administration of isotonic intravenous fluid bolus > 40 mL/kg in 1 hour a decrease in BP (hypotension) <5^th^ percentile for age or systolic BP <2 SD below normal age OR need for vasoactive drug to maintain BP in normal range OR two of the following: unexplained metabolic acidosis (Base deficit >5.0 mEq/L), increased arterial lactate >2 times upper limit of normal, oliguria (urine output <0.5 mL/kg/hr), prolonged capillary refill > 5 sec, core to peripheral temperature gap >3°C.” (Goldstein et al.) | 8 Khan et al.  Kortz et al.  Sharma et al. Santhanam et al.  Ibrahiem et al.  Basnet et al. Sarmin et al.  Chisti et al. |
| Adapted SSC | “Septic shock was defined as sepsis and hypotension (systolic BP<70mmHg in infant; <70+2xage after one year of age) OR sepsis with three of the following four clinical signs of hypoperfusion: decreased pulse volume, capillary filling time < 3 sec, tachycardia (heart rate >190/min in 1 month to 2 years, >140/min in 2 to 8 years and >110/min in >8 years) and urine output <1 mL/kg/hr (<20 mL/hr in >20 kg child)” and “fluid refractory shock was defined as persistence of shock after fluid boluses.” | 4  Upadhyay et al. Chopra et al.  Ramaswamy et al.  Valoor et al. |
| 2. World Health Organization (WHO) Dengue | | |
| WHO Dengue | “Shock is manifested by rapid and weak pulse with narrowing of the pulse pressure (20 mmHg (2.7 kPa) or less) or hypotension, with the presence of cold, clammy skin and restlessness. Dengue Hemorrhagic Fever (DHF) grade III is DHF grade I or II plus circulatory failure. DHF grade IV is as in grade III plus profound shock with undetectable BP and pulse. DHF grade III and IV are defined as Dengue Shock Syndrome (DSS).” | 13  Srivastava et al. Bethell etl al. Dung et al. Nhan et al. Wills et al, 2002 Kabilan et al.  Ranjit et al.  Wills et al, 2005 Pham et al. Kamath et al. Djamiatun et al. Ngwe Tun et al.  Pothapregada et al. |
| 3. WHO ETAT | | |
| WHO ETAT 2016 | “Shock: cold extremities *with* capillary refill > 3 s *and* a weak, fast pulse (all signs must be present)” | 1  Ahmad et al. |
| Adapted WHO | Shock defined as the presence of one or more of the following: conscious level less than alert, capillary refill time of more than 2 seconds, a lower-limb temperature gradient or a weak pulse | 4  Talbert et al.  Webb et al. Maitland et al, 2019  Maitland et al, 2019 |
| Adapted WHO | “We defined shock as two or more signs of capillary refill time > 3 seconds, temperature gradient (cooler extremities to warmer central body to touch) or rapid and weak pulse volume” | 1  Obonyo et al. |
| 4. Other | | |
| Other | “Children with one or more of the following: CRT > 2 seconds, lower limb temperature gradient, weak pulse volume, prolonged capillary refill > 2 seconds, deep ‘acidotic’ or ‘Kussmaul’ breathing, creatinine >80 µmol/L, or depressed conscious state (prostration (inability to sit up if aged > 8 months) if present after correction of hypoglycemia” | 1  Akech et al, 2010 |
| Other | “.. and presented with a severe febrile illness complicated by impaired consciousness (prostration or coma), respiratory distress (increased work of breathing), or both, and with impaired perfusion, as evidenced by one or more of the following: a capillary refill time of 3 or more seconds, lower-limb temperature gradient, weak radial-pulse volume, or severe tachycardia.” | 1  Maitland et al, 2011 |
| Other | “Shock (defined by capillary refilling time > 2 seconds)” | 1  Pedro et al. |
| Other | “Shock was defined as a Base Deficit (BD) >/=6” | 1  Patregnani et al. |
| Other | “To examine the prevalence of shock amongst admissions, shock was defined as a child with any of the following: a clinician’s indication that the child had shock as a problem accompanying diarrhea and dehydration (an indication of the severity of fluid loss); a diagnosis of shock associated with an underlying cause (e.g. septic shock); or use of rapid bolus fluid therapy in a child irrespective of diagnosis” | 1  Mbevi et al.* |
| Other | Clinician documented a diagnosis of shock in medical notes | 1  Akech et al. 2018** |
| Other | “Shock score determined as presence of hypotension or two or more of the following features: hypoxia, tachypnoea, delayed capillary refill, hypothermia, acidosis (and/or deep breathing), elevated creatinine or dehydration (sunken fontanelle and/or sunken eyes). Hypotension was scored as two points; the remaining features as one point each” | 3  Maitland et al, 2003  Maitland et al, 2004  Akech et al, 2006 |
| Other | Shock defined as the presence of low systolic blood pressure, defined as a systolic blood pressure of <50 mmHg, <70 mmHg or less than 5^th^ percentile for age and sex matched controls | 3  Gehlawat et al.  Yadav et al.  Boyce et al. |
| Other | “Decompensated shock (children: systolic blood pressure <70mmHg). Compensated shock (capillary refill > 3 sec/ temperature gradient)” | 2  Kalinga et al.  Dondorp et al. |
| Other | “Children were eligible for inclusion if they had all of the following criteria: Plasmodium falciparum parasitemia, clinical features of severe malaria and metabolic acidosis (a base deficit >8).” “ Shock was defined as non-attainment of all the following resuscitation endpoints: heart rate within appropriate ranges for age, capillary refill time less than 3 seconds, systolic blood pressure within threshold range for age (using a pragmatic cut off of > 70mmHg for children less than 1 year old or >80mmHg if aged >1 year), and oxygen saturations >95% in room air.” | 1  Akech et al. 2010 |
| Other | “Hypovolemic shock was defined as the presence of hypotension, tachycardia, poor peripheral perfusion, low central venous pressure (if catheter was inserted in a central vein), oliguria, and abnormal values of lab tests such as an increase in hematocrit and blood sodium, low urinary sodium and blood protein” | 1  Nguyen et al. |
| 5. No definition | | |
| No definition | - | 6  Baranwal et al. Breurec et al. Kumar et al. Palanivel et al. Narayanasamy et al.  Osifo et al. |

* Mbevi et al. also reported the prevalence of shock using two other definitions of shock.

1. WHO shock + dehydration defined as: “Children fulfilled locally adapted WHO criteria for shock (documented in national clinical policy since 2013) if they had all four of the following: impaired consciousness (AVPU score <V), weak/absent pulse, cold hands and temperature gradient, and capillary refill > 3 seconds, plus sunken eyes and slow skin pinch”
2. ‘Other’ as reported in the table

** Akech et al, 2018 also reported the prevalence of shock using two other definitions of shock.

1. WHO shock + dehydration defined as above
2. Clinical diagnosis of shock made by clinician or fluid bolus given (also if diagnosis of shock was not documented)
