# Supplementary material for: Etiology, Pathophysiology and Mortality of Shock in Children in Low (Middle) Income Countries: A Systematic Review
Source: J Trop Pediatr. 2022 Jul 7;68(4):fmac053. doi: 10.1093/tropej/fmac053 (PMC9586536; doi:10.1093/tropej/fmac053)
Supplement: fmac053_Supplementary_Data [file fmac053_supplementary_data.zip › 20220505 Supplementary Figure 2 Forest plot per region.pptx]

## Slide 1
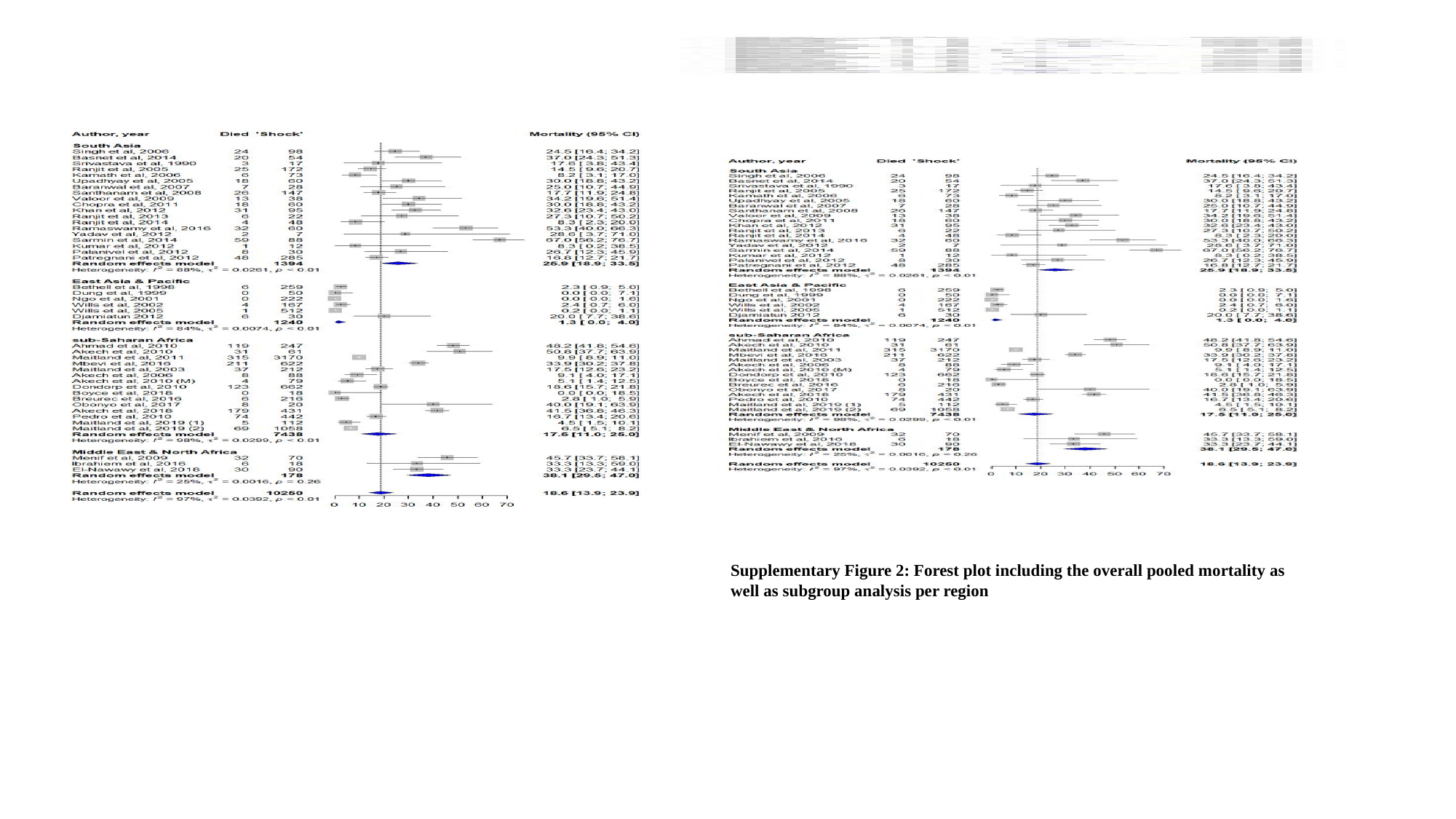

Supplementary Figure 2: Forest plot including the overall pooled mortality as well as subgroup analysis per region
